# An investigation into the performance of the Adjuvant! Online prognostic programme in early breast cancer for a cohort of patients in the United Kingdom

**DOI:** 10.1038/sj.bjc.6605283

**Published:** 2009-09-01

**Authors:** H E Campbell, M A Taylor, A L Harris, A M Gray

**Affiliations:** 1 Department of Public Health, Health Economics Research Centre, University of Oxford, Old Road Campus, Headington, Oxford OX3 7LF, UK; 2 Medical Oncology Unit, Cancer Research UK Oxford Cancer Centre, Churchill Hospital, Headington, Oxford OX3 7LJ, UK

**Keywords:** Adjuvant! Online, prognosis, outcome prediction, performance, breast cancer, adjuvant therapy

## Abstract

**Background:**

Adjuvant! Online is an internet-based computer programme providing 10-year prognosis predictions for early breast cancer patients. It was developed in the United States, has been successfully validated in Canada, and is used in the United Kingdom and elsewhere. This study investigates the performance of Adjuvant! in a cohort of patients from the United Kingdom.

**Methods:**

Data on the prognostic factors and management of 1065 women with early breast cancer diagnosed consecutively at the Churchill Hospital in Oxford between 1986 and 1996 were entered into Adjuvant! to generate predictions of overall survival (OS), breast cancer-specific survival (BCSS), and event-free survival (EFS) at 10 years. Such predictions were compared with the observed 10-year outcomes of these patients.

**Results:**

For the whole cohort, Adjuvant! significantly overestimated OS (by 5.54%, *P*<0.001), BCSS (by 4.53%, *P*<0.001), and EFS (by 3.51%, *P*=0.001). For OS and BCSS, overestimation persisted across most demographic, pathologic, and treatment subgroups investigated. Differences between Adjuvant! predicted and observed EFS appeared smaller, and were significant for far fewer subgroups, only 5 out of the 28. The likely explanation for such discordance is that US breast cancer mortality rates (upon which Adjuvant! is based) appear to be systematically lower than breast cancer mortality rates in the United Kingdom. Differences in survival after recurrence would seem to be one contributory factor, with data suggesting that prognosis after relapse appears poorer in the United Kingdom. This may reflect the fact that new and more effective cancer drugs are often only approved for use in the United Kingdom many years after their adoption in the United States.

**Conclusion:**

The use of Adjuvant! by clinicians within the UK National Health Service is increasing, under the assumption that the programme is transferrable to the United Kingdom. At least for women treated for breast cancer at the Churchill Hospital in Oxford, however, Adjuvant!'s predictions were on the whole overoptimistic. If the findings reported here could be shown to be generalisable to other areas of the United Kingdom, then thought should perhaps be given to the development of a UK-specific version of the programme.

The decision about whether to administer adjuvant systemic therapy to women diagnosed with early invasive breast cancer is complex and requires the synthesis of information on likely prognosis, treatment effectiveness, and patient preferences. Statistical models or programmes that use established prognostic markers to predict outcomes for early breast cancer patients to aid this decision-making process have greatly increased in recent years.

In the United Kingdom, the Nottingham Prognostic Index (NPI) is one of the few prognostic models that have been widely used by clinicians to help inform the selection of women with early breast cancer for adjuvant systemic therapy (Blamey *et al*, 1979; Haybittle *et al*, 1982; Todd *et al*, 1987). On the basis of a simple Cox proportional hazards model and using routinely collected data on tumour stage, size, and grade, the NPI is simple to compute, its predictions demonstrate good discriminative ability, and it has been successfully validated (Todd *et al*, 1987; Galea *et al*, 1992; Sundquist *et al*, 1999). Use of the NPI, however, is somewhat limited with clinicians able only to calculate a patient's index score and then to reference the relevant life table survival curve from a series of prognostic groups constructed by the authors. With the hazard function from the model not ever having been reported, it is not possible to use the NPI in conjunction with estimates of treatment efficacy to generate prognoses for individual patients both before and after any proposed therapy.

Adjuvant! Online (http://www.adjuvantonline.com) is a web-based programme, which is increasingly being used by oncologists in the United Kingdom and which can generate such patient level prognosis predictions (Ravdin *et al*, 2001; Siminoff *et al*, 2006). Developed in the United States and published in 2001, users can input information on a patient's age, oestrogen receptor (ER) status, tumour grade, tumour size, and number of positive nodes, and obtain predictions of 10-year overall survival (OS) (the likelihood of being alive 10 years after the diagnosis of breast cancer was first carried out), breast cancer-specific survival (BCSS) (the likelihood of not dying of breast cancer within 10 years of diagnosis), and event-free survival (EFS) (the likelihood of surviving 10 years without recurrence (local, regional or distant), a second primary breast cancer, or death from breast cancer), both with and without any proposed adjuvant therapy. The performance of Adjuvant! has been evaluated in small cohorts of patients in Germany, and the programme was successfully validated in a large population of Canadian women with early breast cancer in 2005 (Olivotto *et al*, 2005; Euler *et al*, 2006; Schmidt *et al*, 2009). To date, Adjuvant! has not been subjected to a similar validation exercise in the United Kingdom. Given the increasingly widespread use of the programme by clinicians working in the National Health Service (NHS), it is important to assess the performance of the Adjuvant! Online programme by comparing its 10-year predictions with observed outcomes for early breast cancer patients in the United Kingdom. This paper reports the findings from such a study.

## Adjuvant!

For the purpose of this analysis, it is helpful first to understand how the predictions made by Adjuvant! are generated. The programme is based upon data collected from women aged 20–79 years who underwent primary surgery for invasive breast cancer between 1988 and 1992 and who were entered into the US Surveillance, Epidemiology, and End-Results (SEER) tumour registry database (Adjuvant! Online, 2005a). The SEER tumour registry follows approximately 10% of all breast cancer cases in the United States and records data on patient demographics, tumour characteristics, and survival. Since its initial development, Adjuvant! has been updated a number of times so as to incorporate longer term patient follow-up data, more reliable information on cause of death, and the most recent evidence on treatment efficacy. The current version (Version 8) is based upon the observed 10-year survival experiences of women in the tumour registry and robust estimates of treatment effect taken predominantly from the 2005 Early Breast Cancer Trialists' Collaborative Group (EBCTCG) overviews (Early Breast Cancer Trialists' Collaborative Group, 2005).

Users of Adjuvant! can enter information on the following prognostic factors: ER status (positive, negative, undefined), tumour grade (1, 2, 3, undefined), tumour size (0.1–1 cm, 1.1–2 cm, 2.1–3 cm, 3.1–5 cm, >5 cm), and the number of positive nodes (0, 1–3, 4–9, >9). The programme then ‘looks up’ the annual breast cancer mortality rates, which correspond to the combination of prognostic factors entered. The rates returned are those derived from women in the SEER tumour registry with the same combination of specified prognostic factors, and are then subsequently used within an actuarial survival analysis to facilitate patient-level prognosis prediction as follows: a 100% survival probability at time zero (diagnosis) in the model is re-calculated after 1 year as 100% minus the patient's age-adjusted probability of dying from causes other than breast cancer during that year (taken from US life table data), and also minus the patient's expected probability of dying from breast cancer during that year (calculated as described above). From the resulting survival probability at year 1, this process is then repeated again, and annually thereafter, out to 10 years to give an estimate of OS. Breast cancer-specific survival is given by 100% minus the 10-year cumulative probability of dying from breast cancer.

Using this actuarial approach, the principal aim of Adjuvant! is to generate estimates of 10-year outcomes both with and without adjuvant systemic therapy. Estimation of the latter requires that the annual breast cancer mortality rates used reflect the level of risk in the absence of treatment. Some women in the SEER registry would have received adjuvant therapy; however, treatment data were not recorded. It was necessary therefore for analysts developing Adjuvant! to adjust for the expected frequency and benefit of this adjuvant therapy to be able to generate predictions of breast cancer mortality (and ultimately OS and BCSS) ‘without treatment’. The programme was then designed to model the likely effects of any planned adjuvant therapy (hormone therapy, chemotherapy, or both, as specified by the user). As detailed above, the estimates of treatment effect used by Adjuvant! are taken from the published literature. Such relative risks are applied to the programme's ‘without treatment’ breast cancer mortality rates and through the actuarial approach described above, Adjuvant! then provides a prediction of the likely improvement in prognosis (OS and BCSS) offered by adjuvant therapy.

In addition to OS and BCSS, Adjuvant! also provides predictions of EFS. The SEER registry however collects no information on cancer recurrence, and so Adjuvant!'s ‘without treatment’ recurrence probabilities are determined indirectly from its ‘without treatment’ breast cancer mortality predictions (further information is provided in the Discussion section).

## Materials and methods

The study cohort comprised all breast cancer patients diagnosed consecutively between 1986 and 1996 at the Churchill Hospital in Oxford - this hospital operates a regional referral service, receiving patients from across the county of Oxfordshire (total population approximately 635 500) and the surrounding areas (Oxfordshire County Council, 2008). For each woman in the cohort, histopathology reports and treatment record sheets provided information on tumour characteristics and treatments administered. Patients were followed up on an annual basis through the Cancer Intelligence Network and General Practitioners, who returned information on recurrence and survival status.

As Adjuvant! was developed for ‘adjuvant’ decision-making in those where benefit is less certain, from this cohort we excluded women with locally advanced disease (those with T3 (>5 cm tumour) and T4 (tumour of any size growing into the skin or chest wall) tumours, and those with N2 (4–9 nodes involved) and N3 (10 or more lymph nodes involved) tumours), and women with metastatic disease (M1). By excluding such patients, our approach is consistent with that of the Canadian validation study; however, we do acknowledge that these women would still have been considered for and probably would have received systemic hormonal therapy or chemotherapy or both.

To the remaining women in the study cohort, we applied the following eligibility criteria: patients must be aged 85 years or less, have complete data on nodal status, tumour size, and adjuvant systemic therapy, have undergone complete local therapy (that is, radiotherapy given if breast conserving surgery was conducted), and have complete 10-year follow-up.

### Treatment protocol

During 1986 to 1996, the surgical treatment protocol at the Churchill Hospital was for breast conservation followed by radiotherapy, with mastectomy reserved for larger and more central tumours. The sampling of at least four nodes was recommended (median=6, range 1–26) and in the first instance, women found to be node positive and under the age of 60 years were administered adjuvant intravenous CMF chemotherapy. Over time however, as evidence emerged on the effects of adjuvant chemotherapy in broader groups of patients, therapy was extended to more women (e.g., ER-negative older women). Five years of tamoxifen was prescribed for all patients known to have ER-positive tumours. No other factors in addition to the five featured in the Adjuvant! programme were routinely used to select women for adjuvant therapy.

### Data analysis

For each eligible woman, Adjuvant! standard version 8 was used to generate 10-year predictions of OS, BCSS, and EFS. Such predictions were obtained by entering into the programme information on each patient's age, tumour size, number of positive nodes, grade, ER status, and adjuvant systemic therapies received (types of hormone and chemotherapies). In line with the Canadian validation study, all predictions were made with Adjuvant!'s comorbidity assumption set at the default of ‘minor problems’.

Observed 10-year outcomes for each woman were available from the Churchill Hospital data set. Comparisons between predicted and observed outcomes (OS, BCSS, and EFS) were conducted for the whole cohort, and for clinically important subgroups. For each of these separate analyses, Kaplan–Meier survival analysis provided observed 10-year percentages. Predicted 10-year percentages were given by averaging over the relevant Adjuvant! predictions. In line with the Canadian validation study, we considered Adjuvant! reliable enough for clinical use if predicted and observed outcomes were within 2% of one another (Olivotto *et al*, 2005). Statistical uncertainty around these differences was assessed by way of a *t*-test, the statistic for which was calculated by dividing the difference between predicted and observed percentages by s.e. for the observed percentages. A *P*-value of less than 0.05 was used to indicate statistical significance in the first instance. Given the need to perform multiple testing, however, we also later consider a more stringent definition of significance of *P*<0.01.

## Results

Between 1986 and 1996, 1696 women with invasive breast cancer were treated consecutively at the Churchill Hospital in Oxford. From this cohort we excluded 315 (18.6%) women with locally advanced or metastatic disease. Of the remaining 1381 women, 316 (22.9%) were considered ineligible and were excluded from this study for the following reasons: age greater than 85 (*n*=1), unknown tumour size or nodal status (*n*=158), unknown adjuvant systemic therapy (*n*=5), incomplete local therapy (breast conserving surgery without radiotherapy) (*n*=78), and follow-up less than 10 years (*n*=74). This left 1065 women with T1-2, N0, M0 tumours. All 1065 of these patients were available for the analysis of OS, but BCSS and EFS could be estimated for only 1058 patients as a consequence of missing data on relapse status and cause of death. Tables 1, 2 and 3 present patient demographic, pathologic and treatment characteristics, and comparisons between Adjuvant! predicted and observed 10-year OS, BCSS and EFS, respectively.

Considering OS first, Table 1 shows that for nearly all analyses performed, the trends in both predicted and observed OS across different subgroups largely conformed with prior expectation. For example, predicted and observed OS in patients without nodal involvement (80.69 and 75.99%, respectively) were greater than predicted and observed OS in patients with nodal involvement (70.03 and 62.65%, respectively). Similar intuitive trends were observed for tumour grade and tumour size.

Comparing Adjuvant! predicted and observed 10-year OS data shows that Adjuvant! almost continuously overestimated this outcome, and in several cases significantly so. Predicted and observed OS were within 2% of each other only for women with an unknown tumour grade (77.20 *vs* 77.46%, difference=−0.26%) and an ER negative status (71.66 *vs* 69.73, difference=1.93%).

From Table 2, which shows BCSS, it can be seen that both predicted and observed outcomes generally increase with age. Competing risk explains this phenomenon, with older patients much more likely to die from other causes than from breast cancer.

Comparing Adjuvant! predicted and observed 10-year BCSS data showed that, as for OS, predictions made by the online programme are almost all higher than observed patient outcomes. A comparison of the data contained within Tables 1 and 2 reveals that predicted and observed OS and BCSS were significantly different for many of the same subgroups.

Event-free survival is shown in Table 3. As for OS and BCSS, trends in predicted and observed EFS across different clinical subgroups are intuitive. In contrast to OS and BCSS, however, differences between Adjuvant! predicted and observed outcomes appear smaller, and are significant for far fewer subgroups, only 5 out of 28 (excluding 16 patients over age of 76 years). Furthermore, observed outcomes are within 2% of predicted outcomes for six of the subgroup analyses performed.

**Figure 1:**
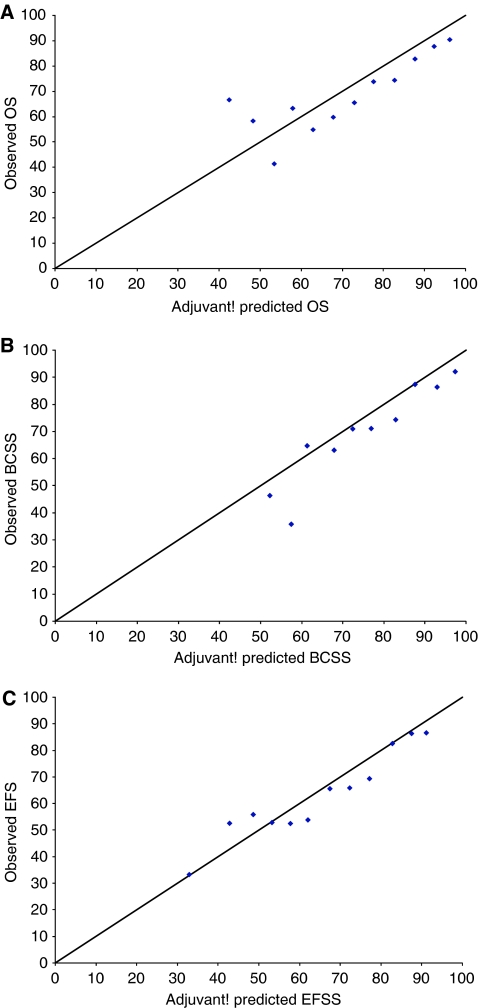
Ten-year Adjuvant! predicted *vs* observed outcomes: (**A**) shows OS, (**B**) shows BCSS, and (**C**) shows EFS.

For the cohort as a whole, Adjuvant!'s predictions for all three outcomes were significantly greater than the observed outcomes. For OS, the difference between predicted and observed was 5.54% (*P*<0.001), for BCSS, 4.53% (*P*<0.001), and for EFS, 3.51% (*P*=0.001). For each outcome we also rank ordered patients on the basis of their Adjuvant! predictions, categorised the cohort into 5% prognosis intervals, and estimated the mean predicted outcome for each group. These mean predictions were then plotted against the observed outcomes for each group as shown in Figure 1.

Figure 1A shows the relationship between predicted and observed OS, and shows that with the exception of three of the groups (where patient numbers were smallest) all points plotted were below the 45 degree line illustrative of perfect agreement between predicted and observed outcomes. Figure 1B, showing BCSS, can be interpreted similarly, however here the magnitude of discrepancy between predicted and observed outcomes was far less consistent across the range of prognoses. Event-free survival is shown in Figure 1C, which illustrates that for five of the groups constructed, Adjuvant!'s predictions were close to observed outcomes.

## Discussion

Adjuvant! Online is a web-based tool from which one can obtain individualised prognosis predictions both with and without adjuvant therapy for women with early breast cancer. Initially developed using data from the SEER registry in the United States, the tool is now used by oncologists to aid clinical decision-making in a number of different countries including Canada, Australia, and the United Kingdom. In 2005, Adjuvant! (version 5.0) was successfully validated in Canada by comparing its 10-year predictions with the actual outcomes of women diagnosed with early breast cancer in British Colombia (Olivotto *et al*, 2005). In that study, which included 4083 patients and informed the format of the analyses presented here, overall predicted and observed 10-year outcomes were within 1% for OS, BCSS, and EFS. For the same demographic, pathologic, and treatment subgroups shown in Tables 1, 2, 3 of this paper, predicted and observed outcomes were almost all within 2%. The authors concluded that Adjuvant! performed reliably in that population. Until now no similar study has examined the performance of Adjuvant! in the United Kingdom.

The data presented in Tables 1, 2, 3 of this paper showed that although both Adjuvant! predictions and observed outcomes generally displayed the expected trends across demographic, pathologic, and treatment subgroups, Adjuvant!'s estimates of outcome were almost consistently (and in some cases significantly) greater than the observed outcomes of the women in the Churchill Hospital data set. In seeking possible explanations for these findings, we focussed first upon our patient cohort, working to establish that they were indeed a representative sample of the wider population of breast cancer patients in the United Kingdom. If patients from Oxfordshire and its surrounding areas were atypical, more specifically if they had higher rates of breast cancer mortality and all-cause mortality than the UK population norm, then this would provide a possible explanation for the findings reported here and at the same time would preclude any generalisation of the results to the rest of the United Kingdom, where Adjuvant! might in fact perform better.

In terms of breast cancer mortality, data from the UK's Office for National Statistics were available to show that the prognosis of breast cancer patients in the Thames Valley Region (which encompasses Oxfordshire and its surrounding counties) is no worse than that of breast cancer patients in England as a whole (Office for National Statistics, 2004a, 2005, 2006a, 2006b). No significant differences existed between country and regional level 5-year age-standardised survival rates for women diagnosed with the disease in 1995–97, 1996–98, and 1997–99. Indeed for patients diagnosed between 1994 and 1996 (the earliest years with data available), 5-year survival in the Thames Valley region was actually better than for England as a whole (77%, 95% CI: 76–79% *vs* 74.9%, 95% CI: 74.5–75.3%) (Office for National Statistics, 2004a).

Similarly, data were available to show that all-cause mortality in Oxfordshire is lower than the UK population norm (Office for National Statistics, 2004b). The Standardised Mortality Ratio (SMR) for the area (a measure comparing actual deaths against expected deaths based upon the mortality rates of the UK population) is 90, thus indicating that local mortality rates are low compared with the national average (an SMR of 100 would have indicated mortality rates equivalent to the UK population norm).

On the basis of these data, one can postulate that the prognoses of the women in our study are likely to be similar, if not slightly better, than those of breast cancer patients in the United Kingdom as a whole. These observations imply that the findings reported here are likely to be representative for the rest of the United Kingdom and so raise the question as to whether there are systematic differences between breast cancer patients in the United States and the United Kingdom, which might explain the optimism of Adjuvant!'s predictions.

**Figure 2:**
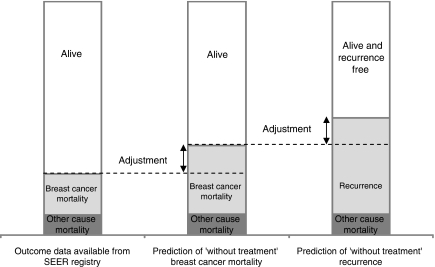
Data and processes used to develop the Adjuvant! Online programme.

In the remainder of the discussion, we attempt to answer this question. Figure 2 (developed by the authors based upon Adjuvant!'s documentation) serves to remind the reader about how Adjuvant! was developed. The first bar in Figure 2 shows the 10-year outcome data available from the SEER registry (survival, and breast cancer and other cause mortality). The second bar depicts how upward adjustments were made to breast cancer mortality probabilities to account for the effects of the adjuvant therapy that women in the SEER registry would likely have received (recall this information was not available). The final bar illustrates how, in the absence of data on recurrence, the probability of experiencing a recurrent event was estimated by applying inflationary factors to the ‘without treatment’ breast cancer mortality probabilities.

We investigated the representativeness of these US-specific data and adjustments for breast cancer patients in the United Kingdom, with a view to ascertaining whether any differences in these parameters exist between the two countries, and if so whether they could potentially explain the overestimation of outcomes seen here. To mirror the way in which Adjuvant! is estimated, the processes involved in estimating breast cancer mortality (bars 1 and 2 in Figure 2) were considered first, followed by breast cancer recurrence (bar 3 in Figure 2). Finally, we considered the US–UK comparability of a number of other factors including life table data (used by Adjuvant! to estimate other cause mortality), tumour staging protocol, and treatment effectiveness.

### Breast cancer mortality

Adjuvant!'s estimates of breast cancer mortality are central to its predictions of both OS and BCSS. The overestimation of BCSS (Table 2) which, recall, is calculated as 100% minus the chance of dying from breast cancer within 10 years of diagnosis, would seem to imply that Adjuvant! is underestimating breast cancer mortality for women in the United Kingdom. The first half of Table 4 further strengthens this hypothesis by presenting predicted and observed BCSS for women in the Churchill Hospital data set who did not receive any adjuvant systemic therapy. Focussing only upon these women provides the opportunity to see how well Adjuvant!'s ‘without treatment’ predictions map to the observed outcomes. As the data show, the programme's estimates of BCSS are predominantly greater than observed BCSS and so by convention its predictions of breast cancer death must be lower.

The two components of Adjuvant!'s ‘without treatment’ breast cancer mortality predictions are the SEER breast cancer mortality data and the inflationary adjustment factors used to remove the effects of unobservable adjuvant therapy (bars 1 and 2 in Figure 2). Considering the inflation factors first, Table 5 gives the values used to adjust the observed breast cancer mortality rates of women in the SEER registry. Inflation factors were estimated for different nodal status/tumour size combinations by multiplying the probability that patients with each particular combination would have received adjuvant therapy by estimates of treatment relative risk reduction taken predominantly from the 2005 EBCTCG Overview (Early Breast Cancer Trialists' Collaborative Group, 2005). The SEER breast cancer mortality rates of women with node-negative tumours 1 cm in diameter for example were inflated by 8%, whereas those of women with three positive nodes and a 5 cm tumour were inflated by 33%. As one would expect, the inflation factors estimated increase as prognosis worsens to reflect the higher expected usage of adjuvant therapy in these women.

If these inflation factors do not accurately reflect the rates of adjuvant therapy administration in the United States between 1988 and 1992, more specifically if they were to underestimate the use of hormone therapy and chemotherapy at that time, then the amount by which the SEER breast cancer mortality rates are inflated would be too small and the resulting ‘without treatment’ breast cancer mortality rates too low, which could potentially explain the overestimation of outcomes observed by this study. Previously published studies on the performance of Adjuvant!, however, indicate that this is not the case (Olivotto *et al*, 2005; Euler *et al*, 2006; Schmidt *et al*, 2009). In the Canadian validation study, for example, Adjuvant!'s predictions of BCSS for subgroups of patients who did not receive adjuvant therapy were almost all within 2% of observed BCSS. This suggests that the programme's estimates of ‘without treatment’ breast cancer mortality are valid, at least for patients in British Columbia (Olivotto *et al*, 2005). Similarly, a study from Germany that included only axillary node-negative breast cancer patients (few of whom received adjuvant CMF), found no significant differences between Adjuvant! predicted and observed OS across patients with complete 10-year follow-up (Euler *et al*, 2006).

These studies would suggest that Adjuvant! can predict breast cancer mortality accurately both in Canada and Germany. Given the results reported in this paper, the question is then whether there are systematic differences in underlying breast cancer mortality rates between the United Kingdom and the United States (as well as Canada and Germany) that mean that the SEER breast cancer mortality rates are not generalisable to patients in the United Kingdom.

**Figure 3:**
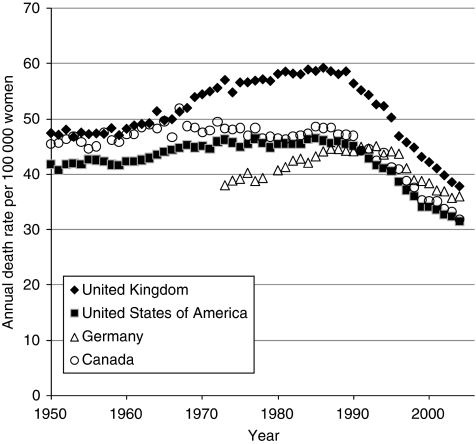
Age-standardised breast cancer mortality in the United States, Canada, Germany, and the United Kingdom for women aged 30–74 years.

Figure 3 plots age-standardised annual breast cancer mortality rates for the United Kingdom, the United States, Canada, and Germany, and shows that from the 1970s onwards, rates in the United Kingdom have been consistently higher than in the other three countries (Cancer Information Section – International Agency for Research on Cancer, 2008). Differences in breast cancer incidence rates do not appear to explain these trends, with the rate of new diagnoses in the United Kingdom similar to that seen in Canada and actually lower than the rate in the United States (Parkin *et al*, 2005). Figure 3 shows the differences in mortality to be particularly pronounced from the 1970s to the start of the 1990s when death rates from breast cancer increased in the United Kingdom but remained relatively stable in the United States and Canada. Although experts have reported that the reasons for the increases in the United Kingdom are unclear, they have cited later childbearing, earlier menarche, and other hormonal influences as potential explanatory factors (Brown, 2000). Whatever the reasons, breast cancer mortality rates appear lower in the United States than in the United Kingdom. On this basis, the application of Adjuvant! Online to breast cancer patients in the United Kingdom might be expected to result in an underestimation of risk and consequently an overestimation of prognosis.

### Recurrence

In addition to OS and BCSS, Adjuvant! also provided predictions of EFS for women in the Churchill data set. Event-free survival is calculated as 100% minus the chance of experiencing a recurrence, a second primary tumour, or death from breast cancer within 10 years of diagnosis. With recurrence as an event not observable from the SEER registry, the recurrence rates used in Adjuvant!'s estimations of EFS are estimated indirectly (bars 2 and 3 in Figure 2). In the first instance, 14% is added to the mortality risk to reflect the fact that not all recurrences will result in breast cancer death. The annual breast cancer mortality rates are then inflated by a factor of 1.6 for women with ER-positive tumours and 1.1 for women with ER-negative tumours (Adjuvant! Online, 2005b).

Given the evidence to show that the breast cancer mortality rates upon which Adjuvant!'s recurrence rates are based could underestimate the level of risk facing breast cancer patients in the United Kingdom, then provided the recurrence inflation factors described above are generalisable to the United Kingdom (i.e., they accurately reflect survival prospects following recurrence in the United Kingdom), one would have expected the magnitude of the overestimation seen for BCSS to have increased further for EFS. To demonstrate (albeit oversimplistically), suppose BCSS as predicted by Adjuvant! and observed in the Churchill Hospital data set were 85 and 80% respectively. Inflating the associated breast cancer mortality rates of 15 and 20% by a fixed factor of say 1.6 (to reflect survival prospects following recurrence) would generate event rates of 24 and 32%, respectively. The resulting EFS rates would be approximately 76 and 68% respectively, and the difference in EFS somewhere in the region of 8% as opposed to the 5% observed for BCSS.

Table 3 however shows that the degree of overprediction for EFS is on average smaller than for BCSS. For the magnitude of Adjuvant!'s overprediction to fall when switching from BCSS as an outcome to EFS, the recurrence rates of women in the Churchill Hospital data set must be closer to their breast cancer mortality rates than is postulated by Adjuvant!'s inflation factors. In effect, the survival prospects of women diagnosed with recurrence at the Churchill Hospital appear poorer than those of their counterparts in the United States. One can speculate as to why this might be the case. It is well known, for example, that clinicians in the United States are more likely to have access to newer and more effective therapies before their counterparts in the United Kingdom. The taxane paclitaxel, for example, was approved by the US Food and Drug Administration (FDA) as a second-line therapy after the failure of anthracyclines for distant recurrence, or for patients relapsing within 6 months of initial breast cancer treatment in 1994 (US Food and Drugs Administration, 2007). In the United Kingdom, however, not until June 2000 was this drug approved by the National Institute for Health and Clinical Excellence for the same indications (National Institute for Health and Clinical Excellence, 2000). Other agents approved for the treatment of recurrent breast cancer in the United States before the United Kingdom, include capecitabine, herceptin, and gemcitabine. Bevacizumab and lapatinib provide examples of drugs currently being used in the United States to treat metastatic breast cancer but not yet approved for use in the United Kingdom. With the approval and licensing of new and more effective breast cancer drugs in the United Kingdom lagging years behind the United States, one might expect there to be differential post-recurrence survival rates between the two countries. In addition, other factors that could influence post-recurrence prognosis and which might conceivably vary between the United States and the United Kingdom include the frequency and intensity of patient follow-up, and patient awareness of the symptoms of recurrence.

### Other factors

In this section, we consider the US–UK comparability of life table data, staging protocol, and treatment effectiveness.

When calculating OS, in addition to the breast cancer mortality rates discussed above, Adjuvant! also makes use of age- and sex-adjusted US life table data to predict other cause mortality. Moving from Table 2, which presents BCSS (calculated as 100% minus the chances of dying from breast cancer within 10 years of diagnosis) to Table 1, which shows OS (given by 100% minus the chances of dying from breast cancer or any other cause within 10 years of diagnosis), one can see that the magnitude of Adjuvant!'s overestimation tends to increase, albeit only slightly, for example, across all patients the difference in BCSS of 4.53% increases to just 5.54% for OS. This still suggests however that there may also be issues surrounding the application of US life table data to UK patients.

A comparison of life table data from the two countries shows that for women below the age of 70 years (92% of women in the Churchill data set), annual mortality risks are virtually identical (National Centre for Health Statistics, 1998; Government Actuary's Department, 2000). For women over the age of 70 years, however, the risk of dying from any cause is lower for women in the United States than in the United Kingdom. An 80-year-old woman in the United States, for example, has a 5% probability of dying before reaching age 81 years. In the United Kingdom, the corresponding figure is 5.6%. Although seemingly small, this difference of 0.6% will culminate in a difference of 6% over 10 years. The effect in the Churchill Hospital data set, however, is far more subtle for a number of reasons. First, only 8% of patients are over the age of 70 years and will therefore be susceptible to this underestimation. Second, women in Oxfordshire have lower all-cause mortality rates than the general UK population (see earlier discussion) and so the survival of older women in the cohort will be closer to that of their US counterparts. Nevertheless, when using Adjuvant! to model the prognosis of older patients, UK users should be aware that the programme will underestimate other cause mortality, and consequently overestimate OS.

With the staging protocol at the Churchill Hospital requiring the resection of at least four axillary lymph nodes, and women in the SEER registry generally having six nodes sampled, the possibility exists that the women in this study may have been understaged, that is, classified as node negative when in fact an examination of a larger number of nodes might have revealed the presence of cancerous deposits. Further investigation of the Churchill data however suggests that this is unlikely to be the case, with no difference in 10-year BCSS seen between patients classified as node negative on the basis of less than four nodes sampled (*n*=112, BCSS=83%, 95% CI 75–89%), four to seven nodes sampled (*n*=309, BCSS=86%, 95% CI 82–90%), and with more than seven nodes sampled (*n*=308, BCSS=84%, 95% CI 79–88%). In addition, although the effect of understaging would manifest for women classified as node negative (the breast cancer mortality within this group would be larger than expected and Adjuvant!'s prognosis predictions for true node-negative patients would then appear overly optimistic), it could by no means explain the discordance between predicted and observed OS and BCSS that is maintained across virtually all demographic, pathologic, and treatment subgroups.

Finally we consider the published estimates of treatment effect used in the Adjuvant! programme to model the likely impact of potential adjuvant therapies. Table 4 shows a comparison of predicted and observed BCSS for women in the Churchill Hospital data set who received some form of adjuvant therapy. Here, and in comparison with women who did not receive adjuvant therapy, Adjuvant!'s predictions are significantly greater than observed outcomes for a much larger number of subgroups. Furthermore, it is interesting to note that for certain subgroups with factors indicative of a poorer prognosis (i.e., younger age, grade 3, positive nodal involvement, and tumour size 2.1–5 cm), the difference between predicted and observed BCSS appears much greater once adjuvant therapies received are entered into the Adjuvant! programme.

A possible explanation for this is that the estimates of treatment effect used by the Adjuvant! programme are taken from a meta analysis of randomised controlled trials (Early Breast Cancer Trialists' Collaborative Group, 2005). Although high in internal validity, the magnitude of the treatment effects reported by such trials are unlikely to be fully replicable within routine practice where lower adherence is likely. Many women (>40%) routinely taking tamoxifen, for example, are known to take ‘drug holidays’ on account of the menopausal symptoms they experience as a result of the drug (Fallowfield, 2005). In contrast although, and despite a more severe toxicity profile, adherence with chemotherapy is likely to be higher, as women must usually present at hospital to receive such treatment intravenously. Although it seems intuitive that lower adherence to ‘self-administered’ adjuvant therapies in routine practice would further augment Adjuvant!'s overpredictions for women in the Churchill Hospital data set receiving such treatment, one must however bear in mind the successful validation of Adjuvant! in Canadian patients receiving the same adjuvant therapies and exposed to the same risk of adverse events (Olivotto *et al*, 2005). The question is then whether there are additional factors in the United Kingdom that mean early breast cancer patients in this country derive less benefit from treatment than their counterparts in Canada and the United States?

### Limitations

This study is not without its limitations. Although when compared with some studies evaluating the performance of Adjuvant!, our study cohort is of a reasonable size, relative to the Canadian validation study, which included over 4000 patients, our sample might be considered small. The issue of sample size is particularly pertinent for the subgroup analyses shown in Tables 1, 2, 3, 4. Although most analyses performed were based upon reasonable numbers of patients, for a few subgroups, most noticeably women aged 20–35 years and 76 years and over, numbers were low, at 34 and 16 patients, respectively. Similarly, categorising the cohort according to prognosis predictions (as seen in Figure 1) also resulted in groups containing small numbers of patients. In both of these cases, the findings presented should be interpreted with caution.

Also related to the subgroup analyses is the issue of multiple hypothesis testing. It is widely accepted that the likelihood of finding a significant difference when one does not truly exist (a Type I error) increases with the number of significance tests performed (Altman, 1999). In this study, where over 30 such tests were performed separately for OS, BCSS, and EFS, one must consider the possibility that some of the differences between predicted and observed outcomes that were significant at the 5% level are in fact spurious. From the published literature it is not clear how best to deal with the issues posed by multiple significance testing. Bonferroni adjustments have been advocated as one means of reducing type I errors; however, this has been shown to be at the expense of increasing type II errors (accepting that there is no difference when in fact the opposite is true). The technique has also been criticised for testing irrelevant hypotheses and leading to inferences which defy common sense (Perenger, 1998). Acknowledging the potential impact of multiple testing in this study, we evaluated our results using a stricter criterion for statistical significance of *P*<0.01, in addition to the conventional level of *P*<0.05. Tables 1 and 2 (OS and BCSS) show that many of the differences significant at the 5% level, remained significant at the 1% level. For EFS, however (Table 3), none of the differences observed were significant at the 1% level.

## Conclusion

Adjuvant! Online is a valuable application capable of generating 10-year prognosis predictions in the absence and presence of adjuvant systemic therapy. The programme was developed in the United States and has been shown to perform well in Canada and Germany. This study however has demonstrated that when applied to a cohort of women treated at Oxford's Churchill Hospital in the United Kingdom, Adjuvant! generated prognosis predictions, which were overoptimistic. A systematic difference in the underlying breast cancer mortality rates between the United States and the United Kingdom would appear to provide the main explanation for why a straightforward application of the programme to a cohort of patients in the United Kingdom appears not to perform as well as expected. Data to suggest that these differences in breast cancer mortality can be partly explained by poorer post-recurrence survival rates in the United Kingdom, also illustrate the wider and more rapid availability of new therapies in the United States than in the United Kingdom.

With clinicians in the United Kingdom increasingly making use of Adjuvant! as an aid to clinical decision making, further research is required to ascertain whether the findings reported here are indeed generalisable to other areas of the United Kingdom. If found to be the case, then one must consider whether certain women may be being advised against adjuvant chemotherapy on the basis of overoptimistic prognosis predictions. Adjustments to the input parameters within the Adjuvant! model so as to produce a UK-specific version could offer one potential solution to this.

## Figures and Tables

**Table 1:** Comparison of Adjuvant! 10-year overall survival predictions with observed 10-year outcomes for 1065 women presenting at the Churchill Hospital in Oxford between 1986 and 1996

	**Patients**		**% Overall survival**			
**Demographic, pathologic, and treatment characteristics**	**No.**	**%**	**Adjuvant! prediction**	**Observed**	**s.e.**	**Difference**
All patients	1065	100	77.37	71.83	1.38	5.54^†^
						
*Age, years*						
20–35	34	3.19	78.74	76.47	7.27	2.27
36–50	363	34.08	82.29	77.96	2.18	4.33^*^
51–65	458	43.00	78.47	74.45	2.04	4.02^*^
66–75	194	18.22	67.32	55.15	3.57	12.17^†^
⩾76	16	1.50	53.11	50.00	12.50	3.11
						
*Menopausal status*						
Pre	397	37.28	81.98	77.83	2.08	4.15^*^
Post	668	62.72	74.62	68.26	1.80	6.36^†^
						
*Histology*						
Ductal	799	75.02	76.99	70.71	1.61	6.28^†^
Lobular	116	10.89	78.25	74.14	4.07	4.11
Other	150	14.08	78.69	76.00	3.49	2.69
						
*Grade*						
1	152	14.27	87.20	83.55	3.01	3.65
2	421	39.53	78.55	71.50	2.20	7.05^†^
3	248	23.29	69.50	59.68	3.11	9.82^†^
Unknown	244	22.91	77.20	77.46	2.68	−0.26
						
*Nodal involvement*						
Negative	733	68.83	80.69	75.99	1.58	4.70^†^
Positive	332	31.17	70.03	62.65	2.65	7.38^†^
						
*Tumour size*						
0.1–1 cm	150	14.08	88.77	82.67	3.09	6.10
1.1–2 cm	471	44.23	82.15	75.58	1.98	6.57^†^
2.1–5 cm	444	41.69	68.45	64.19	2.28	4.26
						
*ER status*						
Negative	261	24.51	71.66	69.73	2.84	1.93
Positive	495	46.48	79.91	70.91	2.04	9.00^†^
Unknown	309	29.01	78.12	75.08	2.46	3.04
						
*Local therapy*						
BCS+RT	822	77.18	78.98	74.94	1.51	4.04^†^
Mast+RT	119	11.17	69.51	58.82	4.51	10.69^*^
Mast, no RT	124	11.64	74.22	63.71	4.32	10.51^*^
						
*Systemic therapy*						
None	252	23.66	77.98	71.83	2.83	6.15^*^
Hormones only	585	54.93	77.30	72.82	1.84	4.48^*^
Chemotherapy only	83	7.79	74.97	66.27	5.19	8.70
Hormones+chemotherapy	145	13.62	77.95	71.03	3.77	6.92

Abbreviations: BCS=breast conserving surgery; ER=oestrogen receptor; Mast=mastectomy; RT=radiotherapy. ^*^P<0.05, ^†^*P*<0.01.

**Table 2:** Comparison of Adjuvant! 10-year breast cancer specific survival predictions with observed 10-year outcomes for 1058 women presenting at the Churchill Hospital in Oxford between 1986 and 1996

	**Patients**		**% Breast Cancer-specific survival**			
**Demographic, pathologic, and treatment characteristics**	**No.**	**%**	**Adjuvant! prediction**	**Observed**	**s.e.**	**Difference**
All patients	1058	100	84.76	80.23	1.25	4.53^†^
						
*Age, years*						
20–35	34	3.21	79.61	78.94	7.08	0.67
36–50	361	34.12	84.69	80.07	2.12	4.62^*^
51–65	454	42.91	85.36	81.85	1.84	3.51
66–75	193	18.24	84.41	75.13	3.81	9.28^*^
⩾76	16	1.51	84.63	91.67	7.98	−7.04
						
*Menopausal status*						
Pre	395	37.33	84.25	79.98	2.03	4.27^*^
Post	663	62.67	85.07	80.31	1.60	4.76^†^
						
*Histology*						
Ductal	793	74.95	84.49	80.43	1.45	4.06^†^
Lobular	115	10.87	85.10	77.25	3.92	7.85^*^
Other	150	14.18	85.97	81.80	3.23	4.17
						
*Grade*						
1	152	14.37	95.00	93.71	2.03	1.29
2	420	39.70	86.18	80.29	1.99	5.89^†^
3	243	22.97	76.01	69.91	3.04	6.10^*^
Unknown	243	22.97	84.66	81.88	2.50	2.78
						
*Nodal involvement*						
Negative	729	68.90	88.43	84.90	1.36	3.53^†^
Positive	329	31.10	76.64	69.91	2.59	6.73^†^
						
*Tumour size*						
0.1–1 cm	148	13.99	95.54	87.59	2.74	7.95^†^
1.1–2 cm	470	44.42	89.80	85.26	1.68	4.54^†^
2.1−5 cm	440	41.59	75.75	72.22	2.20	3.53
						
*ER status*						
Negative	259	24.48	77.36	74.60	2.75	2.76
Positive	491	46.41	88.53	81.91	1.80	6.62^†^
Unknown	308	29.11	84.98	82.24	2.22	2.74
						
*Local therapy*						
BCS+RT	815	77.03	86.01	82.66	1.35	3.35^*^
Mast+RT	119	11.25	75.88	65.91	4.46	9.97^*^
Mast, no RT	124	11.72	85.10	77.89	3.92	7.21
						
*Systemic therapy*						
None	252	23.82	85.37	80.02	2.58	5.35^*^
Hormones only	583	55.10	86.42	82.77	1.61	3.65^*^
Chemotherapy only	83	7.84	77.48	68.90	5.17	8.58
Hormones+chemotherapy	140	13.23	81.08	76.82	3.6	4.26

Abbreviations: BCS=breast conserving surgery, ER=oestrogen receptor; RT=radiotherapy, Mast=mastectomy. ^*^P<0.05, ^†^*P*<0.01.

**Table 3:** Comparison of Adjuvant! 10-year event-free survival predictions with observed 10-year outcomes for 1058 women presenting at the Churchill Hospital in Oxford between 1986 and 1996

	**Patients**		**% Event-free survival**			
**Demographic, pathologic, and treatment characteristics**	**No.**	**%**	**Adjuvant! prediction**	**Observed**	**s.e.**	**Difference**
All patients	1058	100	72.44	68.93	1.46	3.51^*^
						
*Age, years*						
20–35	34	3.21	67.37	67.40	8.08	−0.03
36–50	362	34.22	72.88	67.86	2.47	5.02^*^
51–65	452	42.72	72.91	69.60	2.21	3.31
66–75	194	18.34	71.39	68.53	3.60	2.86
⩾76	16	1.51	72.97	92.31	7.39	−19.34^*^
						
*Menopausal status*						
Pre	396	37.43	72.40	67.81	2.36	4.59
Post	662	62.57	72.47	69.53	1.86	2.94
						
*Histology*						
Ductal	794	75.05	72.28	69.16	1.68	3.12
Lobular	115	10.87	72.54	64.19	4.49	8.35
Other	149	14.08	73.26	71.60	3.76	1.66
						
*Grade*						
1	151	14.27	84.16	86.04	2.90	−1.88
2	421	39.79	73.77	68.89	2.31	4.88^*^
3	244	23.06	64.08	57.60	3.28	6.48^*^
Unknown	242	22.87	71.26	69.63	2.99	1.63
						
*Nodal involvement*						
Negative	727	68.71	75.84	73.64	1.67	2.20
Positive	331	31.29	64.98	58.57	2.78	6.41^*^
						
*Tumour size*						
0.1–1 cm	148	13.99	83.94	80.09	3.31	3.85
1.1–2 cm	469	44.33	77.85	74.10	2.08	3.75
2.1–5 cm	441	41.68	62.83	59.47	2.40	3.36
						
*ER status*						
Negative	260	24.57	67.09	61.79	3.06	5.30
Positive	491	46.41	76.57	72.70	2.08	3.87
Unknown	307	29.02	70.38	68.97	2.7	1.41
						
*Local therapy*						
BCS+RT	816	77.13	73.91	71.50	1.61	2.41
Mast+RT	119	11.25	63.29	57.32	4.65	5.97
Mast, no RT	123	11.63	71.60	62.71	4.61	8.89
						
*Systemic therapy*						
None	252	23.82	69.64	67.32	3.03	2.32
Hormones only	582	55.01	74.25	72.54	1.91	1.71
Chemotherapy only	83	7.84	67.53	59.12	5.48	8.41
Hormones+chemotherapy	141	13.33	72.91	62.70	4.10	10.21^*^

Abbreviations: BCS=breast conserving surgery, ER=oestrogen receptor; Mast=mastectomy, RT=radiotherapy. None of the differences observed for EFS achieved significance in the *P*<0.01 level, ^*^P<0.05.

**Table 4:** Comparison of Adjuvant! 10-year predictions and observed breast cancer-specific survival for women presenting at the Churchill Hospital in Oxford between 1986 and 1996 and who did and did not receive adjuvant systemic therapy

	**Patients**		**% Breast cancer-specific survival without adjuvant systemic therapy**				**Patients**		**% breast cancer-specific survival with some adjuvant systemic therapy**			
**Demographic, pathologic, and treatment characteristics**	**No.**	**%**	**Adjuvant! prediction**	**observed**	**s.e.**	**Difference**	**No.**	**%**	**Adjuvant! prediction**	**Observed**	**s.e.**	**Difference**
All patients	252	100	85.37	80.02	2.58	5.35^*^	806	100	84.57	80.31	1.43	4.26^†^
												
*Age, years*												
⩽50	112	44.44	83.96	80.95	3.74	3.01	283	35.11	84.36	79.59	2.41	4.77^*^
51–65	91	36.11	86.74	79.89	4.24	6.85	363	45.04	85.02	82.35	2.04	2.67
⩾66	49	19.44	86.05	76.82	6.90	9.23	160	19.85	83.93	75.93	3.70	8.0^*^
												
*Menopausal status*												
Pre	112	44.44	83.96	80.95	3.74	3.01	283	35.11	84.36	79.59	2.41	4.77^*^
Post	140	55.56	86.50	79.20	3.58	7.30^*^	523	64.89	84.69	80.61	1.79	4.08^*^
												
*Grade*												
1–2	112	44.44	89.60	82.42	3.67	7.18	460	57.07	88.26	84.21	1.75	4.05^*^
3	53	21.03	76.00	71.33	6.49	4.67	190	23.57	76.01	69.59	3.44	6.42
Unknown	87	34.52	85.63	82.04	4.21	3.59	156	19.35	84.11	81.78	3.12	2.33
												
*Nodal involvement*												
Negative	241	95.63	86.24	80.52	2.61	5.72^*^	488	60.55	89.51	87.09	1.56	2.42
Positive	11	4.37	66.28	68.59	15.15	−2.31	318	39.45	77.00	69.95	2.63	7.05^†^
												
*Tumour size*												
0.1–2 cm	158	62.70	91.85	82.88	3.06	8.97^†^	460	57.07	90.95	86.84	1.61	4.11^*^
2.1–5 cm	94	37.30	74.48	75.10	4.62	−0.62	346	42.93	76.10	71.45	2.50	4.65
												
*ER status*												
Negative	59	23.41	80.22	75.09	5.77	5.13	200	24.81	76.52	74.51	3.12	2.01
Positive	67	26.59	90.03	75.98	5.44	14.05^*^	424	52.61	88.30	82.84	1.90	5.46^†^
Unknown	126	50.00	85.30	84.44	3.29	0.90	182	22.58	84.75	80.80	2.96	3.95

Abbreviation: ER=oestrogen receptor.

^*^P<0.05, ^†^*P*<0.01.

**Table 5:** Estimates of average treatment relative risk used by Adjuvant! to adjust for unobserved adjuvant therapy

**Group (nodal status/tumour size)**	**Average relative risk reduction used by Adjuvant! (%)**
(1) (Negative/⩽1 cm)	8
(2) (Negative/1.1–2 cm)	16
(3) (Negative/2.1–5 cm)	33
(4) (1–3 Positive/⩽2 cm)	33
(5) (1–3 Positive/2.1–5 cm)	33
